# Age-dependent visual exploration during simulated day- and night driving on a motorway: a cross-sectional study

**DOI:** 10.1186/s12877-015-0015-2

**Published:** 2015-02-28

**Authors:** Prabitha Urwyler, Nicole Gruber, René M Müri, Michael Jäger, Rahel Bieri, Thomas Nyffeler, Urs P Mosimann, Tobias Nef

**Affiliations:** Gerontechnology and Rehabilitation Group, University of Bern, Murtenstrasse 50, 3010 Bern, Switzerland; Departments of Neurology and Clinical Research, Perception and Eye Movement Laboratory, University Hospital Inselspital, University of Bern, 3010 Bern, Switzerland; Center of Neurology and Neurorehabilitation, Luzerner Kantonsspital, Spitalstrasse, 6000 Luzern 16, Switzerland; University Hospital of Old Age Psychiatry, University of Bern, Murtenstrasse 21, 3010 Bern, Switzerland; ARTORG Center for Biomedical Engineering Research, University of Bern, Murtenstrasse 50, 3010 Bern, Switzerland

**Keywords:** Visual exploration, Daylight driving, Night driving, Age, Fixation durations, Regions of interest, Gaze behavior, Simulated driving

## Abstract

**Background:**

Central and peripheral vision is needed for object detection. Previous research has shown that visual target detection is affected by age. In addition, light conditions also influence visual exploration. The aim of the study was to investigate the effects of age and different light conditions on visual exploration behavior and on driving performance during simulated driving.

**Methods:**

A fixed-base simulator with 180 degree field of view was used to simulate a motorway route under daylight and night conditions to test 29 young subjects (25–40 years) and 27 older subjects (65–78 years). Drivers’ eye fixations were analyzed and assigned to regions of interests (ROI) such as street, road signs, car ahead, environment, rear view mirror, side mirror left, side mirror right, incoming car, parked car, road repair. In addition, lane-keeping and driving speed were analyzed as a measure of driving performance.

**Results:**

Older drivers had longer fixations on the task relevant ROI, but had a lower frequency of checking mirrors when compared to younger drivers. In both age groups, night driving led to a less fixations on the mirror. At the performance level, older drivers showed more variation in driving speed and lane-keeping behavior, which was especially prominent at night. In younger drivers, night driving had no impact on driving speed or lane-keeping behavior.

**Conclusions:**

Older drivers’ visual exploration behavior are more fixed on the task relevant ROI, especially at night, when driving performance becomes more heterogeneous than in younger drivers.

## Background

Vision plays a significant role in driving performance. Visual acuity, contrast sensitivity, glare, visual fields, color vision, night vision, motion perception and dynamic visual acuity are all important for being able to successfully perform the driving task. With increasing age, a myriad of changes occur in the vision system: the pupil becomes more constricted and less able to dilate under low light conditions, and the integrity of the macular pigment and neural pathways is altered [1]. These changes lead to decreased light sensitivity, increased glare sensitivity, reduced visual acuity, and prolonged dark adaptation [1,2]. Therefore, older people need much more light to achieve the same level of retinal illumination as a younger person [3].

In addition, changes in illumination induce physiological changes in the eye that affect vision. The different levels of ambient luminance are: scotopic (lower than 10^−3^ cd/m2); mesopic (10^−3^ to 10 cd/m2) and photopic (above 10 cd/m2) [4]. Scotopic vision is necessary for pitch-black or very low light levels, and is dominated by the use of rods located in the fovea. Photopic vision refers to vision under well-lit conditions, whereas mesopic vision is a combination of both photopic and scotopic vision active in low but not quite dark lighting situations. Night driving requires mesopic rather than scotopic vision, because there is still residual light available when driving at night. Mesopic vision uses both rods and cones of the retina. Reduced light conditions during night driving worsen the visual perception with a greater impact on older drivers than younger drivers [5]. The visual abilities of older adults’ are taxed at low luminance due to loss of retinal rod sensitivity, slower dark adaptation and slower glare recovery [6,7]. With increasing age, mesopic vision decreases and glare sensitivity increases, hence, older drivers often report visual difficulties during night driving, even in the absence of ocular diseases [8]. With the increasing number of elderly drivers, more and more drivers are experiencing night vision difficulties. Several studies have showed the effect of age and light condition on vision and how specific visual functions are correlated to driving performance (for reviews see e.g. [9-11]).

Visual search, distribution of visual attention, target detection and risk perception are crucial elements while driving and can be studied by analyzing the visual exploration behavior ( i.e. eye movements, scanning behavior, gaze fixations, fixation durations). The fovea is the center of highest visual acuity, however foveal vision only covers a small part of the visual scene [12]. Consequently, eye movements are needed to explore the scene to find the most relevant information [13]. In a previous study, we showed that the detection of targets in a visual search task decreases with age, especially for peripheral targets [14]. Several studies examined visual exploration during specific driving tasks such as curve driving [15,16], lane change [17], intersections [18,19], intentional car following [20], and to compare the performance of novice drivers with experienced drivers [15,21-23]. Few studies, however, have assessed age-dependent effects of visual exploration during driving [18,24]. Maltz et al. [24] showed that older experienced drivers focus on a smaller subset of areas when viewing traffic scene images, whereas visual exploration in younger experienced drivers is more evenly distributed. Age-related differences in visual exploration behavior have also been shown at intersections with older drivers scanning significantly less toward the left and right during intersection negotiations when compared to middle-aged and young drivers [18]. During night driving, Crundall et al. [20] found that the horizontal visual exploration behavior is reduced during intentional car following.

In addition to visual exploration behavior, driving performance can also be affected by age. Older drivers drive slower [25-27] and have a more variant speed and lane behavior [28]. Moreover, light conditions also influence driving performance. Average speed has been found to decrease with reduced illumination [26]. At night, older drivers have difficulties with sign recognition, road edge excursions, maintaining appropriate driving speeds and steering accuracy [29]. Thus, it is important to understand the impact of age on night diving and driving performance where visual abilities further decline with night luminance levels.

Existing studies have focused mainly on specific driving situations (e.g. intersections, lane change, curve driving, intentional car following) [15-20], and there is a lack of studies looking at free driving. In addition, little is known about age-dependent visual exploration during night driving. Therefore, the aim of the study was to investigate the effects of age and light conditions on visual exploration behavior during simulated day and night driving. We hypothesized that (i) visual exploration behavior depends on age; (ii) older drivers focus on a smaller area than younger drivers; (iii) older drivers focus more on central areas (street) while neglecting peripheral regions (environment, mirrors); (iv) visual exploration behavior narrowed during simulated night driving. As a second aim, we investigated whether age and light conditions have an effect on driving performance. Regarding lane-keeping and driving speed, we expected a worse lane behavior and lower driving speed for older drivers than younger drivers. In our study, we had four types of driving tasks for both light conditions.

## Methods

### Participants and demographic data

Twenty-nine healthy young participants (age = 31.5 years, SD = 4.2 years, age range = 25-40, mean driving experience since passing test = 12.0 years, SD = 4.6 years, mean weekly mileage = 247.5 km, SD = 282.3 km) and 27 healthy older participants (age = 70.3 years, SD = 3.8 years, age range = 65-78, mean driving experience since passing test = 46.8 years, SD = 7.1 years, mean weekly mileage = 128.4 km, SD = 112.1 km) were recruited from the University of Bern and advertisement in local newspapers. This study was carried out in accordance with the latest version of the Declaration of Helsinki and was approved by the local ethics commission of the Canton Bern, Switzerland. Written informed consent was obtained from all participants prior to the experiment. After filling out a medical history questionnaire that focused on past or current eye disease, participants were screened for visual impairments as well as for cognitive impairment. Far visual acuity (5 m test distance) was measured using Landolt rings [30], mesopic visual acuity and glare sensitivity using the Mesotest II (Oculus, Germany), and binocular contrast sensitivity using the Pelli-Robson contrast sensitivity chart (1 m test distance, [31]). Cognition was assessed using the Montreal Cognitive Assessment (MoCA) [32], the Trail Making Test (TMT) A and B [33], and the Clock Drawing Test (CDT) [34]. Mobility was assessed using the Timed up & go test [35]. The driving habits questionnaire (DHQ) was used to evaluate the participants driving experience and limitations [36]. The inclusion criteria for the study were normal or corrected-to-normal far visual acuity of 0.6 or higher, MoCA ≥ 26, driving experience ≥ 5 years, and no manifest eye disease. Three participants were excluded due to cognitive or visual impairment.

### Apparatus and driving scene

**Figure 1:**
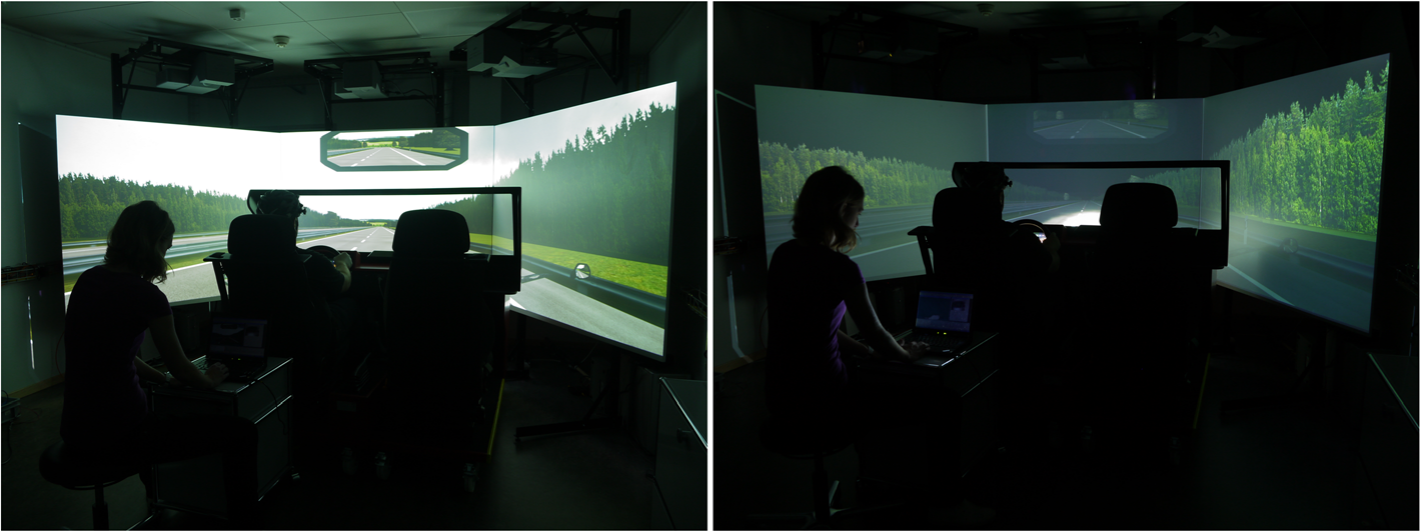
**Driving simulator visual imagery during the day** **(left)** **and night** **(right)** **scenario.**

A fixed-base driving simulator with a partial car cab (F12PI-3/A88, Foerst GmbH, Wiehl, Germany) was used to measure simulated driving performance. The simulator car was mounted with instruments of a Ford Focus. The driving scene was projected on three projection screens (1.80 × 1.39 m), realizing a 180° horizontal and 40° vertical field of view [37]. A two-lane motorway was used as a driving route. It included a straight section with two wide left-hand curves and three wide right-hand curves. The scenery around the motorway was characterized by a landscape with forests and an on-ramp. Figure 1 shows the driving simulator visual imagery during the day and night condition. The luminance levels of the different imagery under day (center screen street 30.5 cd/m^2^, left screen street 31.8 cd/m^2^, grass 22.6 cd/m^2^, trees 8.0 cd/m^2^, sky 136.5 cd/m^2^) and night conditions (center screen street 3.7 cd/m^2^, left screen street 0.79 cd/m^2^, grass 0.56 cd/m^2^, trees 0.54 cd/m^2^, sky 0.46 cd/m^2^) were recorded using a luminance meter (LS-110, Minolta Co. Ltd., Osaka, Japan). Each subject drove once under daylight condition and once under night condition in randomized order. Both driving scenes included the same route, but with the following sub-tasks in randomized order for day and night conditions: drive on a straight stretch, navigate along a narrow lane past the road repair section, three cars to overtake, and avoid collision with a parked car between the service lane and the right lane. There was no other ambient traffic to ensure a standardized driving scene for all subjects. Speed limit, indicated by street signs, was 80 km/h with the exception of 60 km/h along the roadwork section. The entire driving route was 5.9 km long and took participants five to six minutes to complete. To become familiar with the driving simulator, all participants drove a five minutes practice route on a motorway under daylight condition without any other vehicles. After this training section, three participants felt slightly discomfort most likely due to simulator sickness. They were excluded from further analysis. Participants were instructed to drive as they would normally do and follow traffic regulations and traffic signs.

Eye movements of all subjects were recorded using a SMI iView X HED, 50Hz video-based/corneal reflection tracker using a five-point calibration scheme (SensoMotoric Instruments GmbH, Teltow, Germany). Four participants were excluded from the visual exploration analysis due to poor eye video quality.

### Statistical analysis

Visual fixations were detected using a dispersion-based algorithm with a minimum fixation duration of 100 ms and a maximum dispersion of two degrees [17]. After that, mean fixation durations were calculated. Outliers were identified using Tukey’s method and values outside the threefold inter-quartile range were excluded (Young: 614 outliers out of 35,928 fixations (1.70%), Older: 754 outliers out of 28,048 fixations (2.69%)) from analysis [38].

Fixation locations were analyzed over the entire driving route using the video output of the eye-tracking camera. The system generated a scene video with an overlaid gaze cursor representing subject’s visual fixation. Twelve regions of interest (ROI: street, road signs, car ahead, incoming car, parked car, road repair, environment, dashboard, rear view mirror, side mirror left, side mirror right, others) were defined and visual fixations were assigned to these ROIs. For all sub-tasks, relevant ROIs were street, road signs, rear view mirror, side mirror left, side mirror right, while environment was considered as non-relevant ROI. Additional relevant ROIs were road repair for the narrow lane driving task, parked car for the avoid collision task and car ahead and incoming car for the overtaking task.

For each ROI, the ROI DWELL was calculated as the cumulated fixation duration divided by the total driving task duration. Therefore, ROI DWELL is expressed in percentages of the total driving task duration to account for the individual driving task duration [39]. In addition, ROI GAZE DURATION (in seconds) was calculated and defined as the average fixation time spent on ROIs before moving to another ROI.

Lane-keeping precision and driving speed were analyzed as an output of the driving simulator setup. A straight section, where no lane change was required, was used for the driving speed and lane-keeping analysis to ensure the same conditions for all participants (speed limit 80 km/h, no other vehicles).

Group differences were calculated using the independent t-test for parametric data or the Mann–Whitney U-Test for non-parametric data. To calculate the effect of age, light and the interaction of light x age conditions, a 2x2 mixed ANOVA was performed with age group (young, old) as between-subjects independent variable and light conditions (day, night) as the repeated measure within-subject variable. Effect sizes are reported using partial η^2^. Sphericity was tested with a Mauchly-Test and homogeneity of variance was tested using Levene’s Test. If sphericity was violated, a Greenhouse-Geisser correction was applied. A p-value < .05 was considered statistically significant, and the reported p-values are two-sided. SPSS Software (Version 20) was used for statistical analysis.

## Results

### Demographics

The demographics of the 29 younger and 27 older drivers included in the analysis are summarized in Table 1. One of the included older drivers had a strategic choice not to drive at night within the last three months, while the 95.24% of older drivers drove at night. There was no group difference in cognitive function as measured using the MoCA score and the clock drawing test. Older drivers had significantly more driving experience compared to younger drivers. It is also shown in Table 1 that older drivers had a worse far visual acuity and a worse contrast sensitivity as compared to younger drivers. Table 1 further shows that a higher percentage of younger drivers could discriminate a lower level of contrast under mesopic light condition than older drivers, both in presence or absence of glare.

**Table 1:** **Demographics and Clinical parameters of the subjects**

**Demographics/** **Clinical variable**	**Young** **(N = ** **29)**	**Old** **(N = ** **27)**	**Significance**
Age in years [mean ± SD]	31.5 ± 4.2	70.3 ± 3.8	t(54) = −35.951, p < 0.001
MoCA score [mean ± SD]	29.2 ± 1.0	28.5 ± 1.4	U = 286.0, p = 0.069
Trail making test A (sec) [mean ± SD]	21.7 ± 5.6	36.9 ± 12.7	U = 73.5, p < 0.001
Trail making test B (sec) [mean ± SD]	45.3 ± 13.6	84.4 ± 31.4	t(54) = −5.962, p < 0.001
Clock drawing test [mean ± SD]	6.9 ± 0.4	6.6 ± 0.9	ns
Timed up & Go (sec) [mean ± SD]	7.4 ± 0.8	7.8 ± 0.8	t(54) = −2.048, p = 0.045
Years of driving experience [mean ± SD]	12.0 ± 4.6	46.8 ± 7.1	t(54) = −21.935, p < 0.001
Weekly mileage [mean ± SD] [range]	247.5 ± 282.3 [4 - 1186]	128.4 ± 112.1 [8 – 466]	ns
Best far visual acuity [mean ± SD]	1.16 ± 0.13	0.93 ± 0.25	U = 187.5, p < 0.001
Contrast sensitivity (binocular) [mean ± SD]	1.95 ± 0.00	1.91 ± 0.11	U = 333.5, p = 0.033
Mesopic visual acuity [number (%)]			Χ^2^(4) = 7.996, p = 0.092
Contrast 1:2	28 (96.6)	19 (70.4)	
Contrast 1:2.7	0 (0)	3 (11.1)	
Contrast 1:5	1 (3.4)	2 (7.4)	
Contrast 1:23	0 (0)	1 (3.7)	
Nothing seen	0 (0)	2 (7.4)	
Glare sensitivity [number (%)]			Χ^2^(4) = 24.059, p < 0.001
Contrast 1:2	29 (100)	11 (40.7)	
Contrast 1:2.7	0 (0)	5 (18.5)	
Contrast 1:5	0 (0)	4 (14.8)	
Contrast 1:23	0 (0)	1 (3.7)	
Nothing seen	0 (0)	6 (22.2)	

MoCA: Montreal Cognitive Assessment; ns: not signifcant.

### Visual exploration behavior

Both age (F(1,44) = 6.5, p = 0.015, η^2^ = 0.187) and light (F(1,44) = 47.4, p < 0.001, η^2^ = 0.524) had a significant main effect on mean fixation duration. Significantly longer mean fixation durations were found for older drivers (Day vs. Night: 288 ± 43 vs. 308 ± 45) compared to younger drivers (Day vs. Night: 262 ± 35 vs. 276 ± 36) for both day and night conditions, while mean fixation durations were longer at night for both age groups.

#### Driving on a straight stretch

Light had a significant effect on the DURATION for ROI side mirror right and DWELL for ROI rear view mirror, while age had a significant effect on the DURATION for ROI street and DWELL for ROI environment and rear view mirror as shown in Table 2. Older drivers focused longer on the street, while younger drivers focused more on the environment and the rear view mirror while driving on a straight stretch. The frequency of rear view mirror usage and the duration of side view mirror usage was reduced for both age groups at night.

**Table 2:** **Comparison of DWELL and GAZE DURATION in addition to effects of age**, **light and interaction of age x light while driving on a straight stretch**

		**DWELL** **[%]** **(SD)**					**DURATION** **[s]** **(SD)**				
		**Young**	**Old**	**Age**	**Light**	**Age x light**	**Young**	**Old**	**Age**	**Light**	**Age x light**
**Street**	Day	48.2 (8.5)	55.5 (11.7)	F(1,43) = 3.0 p = 0.090 η^2^ = 0.085	F(1,43) = 1.5 p = 0.229 η^2^ = 0.033	F(1,43) = 1.0 p = 0.330 η^2^ = 0.022	0.8 (0.3)	1.1 (0.4)	F(1,43) = 7.7 p = 0.008 η^2^ = 0.152	F(1,43) = 3.1 p = 0.085 η^2^ = 0.068	F(1,43) = 0.2 p = 0.682 η^2^ = 0.004
	Night	52.7 (14.3)	55.9 (13.7)				1.0 (0.4)	1.2 (0.6)			
**Road signs**	Day	18.5 (6.3)	17.4 (12.2)	F(1,43) = 0.3 p = 0.616 η^2^ = 0.006	F(1,43) = 2.2 p = 0.146 η^2^ = 0.049	F(1,43) = 0.0 p = 0.899 η^2^ = 0.000	0.6 (0.2)	0.7 (0.4)	F(1,43) = 2.3 p = 0.138 η^2^ = 0.050	F(1,43) = 1.6 p = 0.222 η^2^ = 0.034	F(1,43) = 0.1 p = 0.742 η^2^ = 0.003
	Night	20.9 (9.3)	19.5 (10.1)				0.7 (0.3)	0.8 (0.2)			
**Environment**	Day	4.2 (5.0)	1.5 (1.9)	F(1,43) = 5.3 p = 0.026 η^2^ = 0.110	F(1,43) = 0.0 p = 0.873 η^2^ = 0.001	F(1,43) = 0.9 p = 0.363 η^2^ = 0.019	0.5 (0.3)	0.3 (0.2)	F(1,43) = 2.5 p = 0.118 η^2^ = 0.056	F(1,43) = 0.1 p = 0.740 η^2^ = 0.003	F(1,43) = 2.9 p = 0.095 η^2^ = 0.063
	Night	3.2 (4.3)	2.2 (3.9)				0.4 (0.4)	0.4 (0.4)			
**Rear** **-view mirror**	Day	6.0 (4.6)	3.3 (5.1)	F(1,43) = 6.3 p = 0.015 η^2^ = 0.129	F(1,43) = 6.8 p = 0.013 η^2^ = 0.136	F(1,43) = 0.2 p = 0.696 η^2^ = 0.004	0.6 (0.3)	0.3 (0.4)	F(1,43) = 3.6 p = 0.066 η^2^ = 0.076	F(1,43) = 0.6 p = 0.445 η^2^ = 0.014	F(1,43) = 4.1 p = 0.050 η^2^ = 0.086
	Night	3.9 (3.3)	1.8 (2.3)				0.5 (0.4)	0.5 (0.5)			
**Side mirror left**	Day	0.1 (0.3)	0.1 (0.7)	F(1,43) = 2.6 p = 0.696 η^2^ = 0.004	F(1,43) = 3.7 p = 0.062 η^2^ = 0.078	F(1,43) = 0.1 p = 0.775 η^2^ = 0.002	0.1 (0.1)	0.1 (0.2)	F(1,43) = 0.1 p = 0.795 η^2^ = 0.002	F(1,43) = 3.8 p = 0.059 η^2^ = 0.081	F(1,43) = 0.5 p = 0.473 η^2^ = 0.012
	Night	0.0 (0.0)	0.1 (0.3)				0.0 (0.0)	0.02 (0.1)			
**Side mirror right**	Day	1.7 (2.1)	1.4 (1.5)	F(1,43) = 0.9 p = 0.357 η^2^ = 0.020	F(1,43) = 1.3 p = 0.265 η^2^ = 0.029	F(1,43) = 0.1 p = 0.769 η^2^ = 0.002	0.4 (0.4)	0.4 (0.4)	F(1,43) = 0.0 p = 0.835 η^2^ = 0.001	F(1,43) = 4.5 p = 0.040 η^2^ = 0.094	F(1,43) = 0.35 p = 0.558 η^2^ = 0.008
	Night	1.4 (2.3)	0.9 (2.2)				0.3 (0.3)	0.2 (0.4)			

DWELL = cumulated fixation duration/total driving task duration; GAZE DURATION = average fixation time; η^2^ refers to partial η^2^.

#### Driving on a narrow lane along a road repair

Light had a significant effect on the visual exploration of ROI road repair and side view mirror, while age had a significant effect on ROI rear view mirror as shown in Table 3. Younger drivers focused more and longer on rear view mirror during this task. Both age groups focused more and longer at the road repair during night time. The usage of side mirror dropped for both age groups at night. However, an interaction of light x age had a significant effect on the ROI DURATION of side view mirror.

**Table 3:** **Comparison of DWELL and GAZE DURATION in addition to effects of age**, **light and interaction of age x light while driving on a narrow lane**

		**DWELL** **[%]** **(SD)**					**DURATION** **[s]** **(SD)**				
		**Young**	**Old**	**Age**	**Light**	**Age x light**	**Young**	**Old**	**Age**	**Light**	**Age x light**
**Street**	Day	48.4 (11.0)	54.4 (11.9)	F(1,43) = 1.3 p = 0.259 η^2^ = 0.030	F(1,43) = 3.5 p = 0.067 η^2^ = 0.076	F(1,43) = 1.5 p = 0.230 η^2^ = 0.033	1.1 (0.4)	1.4 (0.4)	F(1,43) = 3.0 p = 0.089 η^2^ = 0.066	F(1,43) = 2.5 p = 0.124 η^2^ = 0.054	F(1,43) = 1.3 p = 0.262 η^2^ = 0.029
	Night	47.2 (12.6)	48.7 (14.5)				1.3 (0.4)	1.4 (0.6)			
**Road signs**	Day	6.8 (4.0)	4.9 (3.7)	F(1,43) = 3.4 p = 0.069 η^2^ = 0.075	F(1,43) = 0.1 p = 0.806 η^2^ = 0.001	F(1,43) = 0.1 p = 0.819 η^2^ = 0.001	1.4 (0.6)	0.8 (0.5)	F(1,40) = 3.0 p = 0.092 η^2^ = 0.070	F(1,40) = 0.2 p = 0.678 η^2^ = 0.004	F(1,40) = 2.8 p = 0.100 η^2^ = 0.066
	Night	6.4 (4.8)	4.9 (4.4)				1.2 (0.7)	1.1 (0.9)			
**Road repair**	Day	22.2 (6.7)	21.1 (9.0)	F(1,43) = 0.7 p = 0.409 η^2^ = 0.016	F(1,43) = 14.7 p < 0.001 η^2^ = 0.255	F(1,43) = 2.8 p = 0.099 η^2^ = 0.062	0.8 (0.2)	0.9 (0.2)	F(1,43) = 1.8 p = 0.192 η^2^ = 0.029	F(1,43) = 7.8 p = 0.008 η^2^ = 0.606	F(1,43) = 0.3 p = 0.606 η^2^ = 0.006
	Night	26.1 (10.4)	31.2 (13.6)				1.0 (0.2)	1.1 (0.7)			
**Environment**	Day	1.1 (2.4)	0.4 (0.7)	F(1,43) = 0.1 p = 0.828 η^2^ = 0.001	F(1,43) = 0.0 p = 0.860 η^2^ = 0.001	F(1,43) = 1.8 p = 0.191 η^2^ = 0.039	0.3 (0.3)	0.2 (0.3)	F(1,41) = 0.0 p = 0.932 η^2^ = 0.000	F(1,41) = 3.2 p = 0.080 η^2^ = 0.069	F(1,41) = 1.3 p = 0.267 η^2^ = 0.029
	Night	0.4 (1.1)	1.4 (5.4)				0.1 (0.2)	0.2 (0.4)			
**Rear** **-view mirror**	Day	3.6 (2.8)	1.4 (2.0)	F(1,43) = 13.4 p = 0.001 η^2^ = 0.237	F(1,43) = 3.4 p = 0.074 η^2^ = 0.072	F(1,43) = 0.8 p = 0.387 η^2^ = 0.017	0.7 (0.3)	0.4 (0.4)	F(1,43) = 7.3 p = 0.010 η^2^ = 0.145	F(1,43) = 2.6 p = 0.116 η^2^ = 0.056	F(1,43) = 1.5 p = 0.224 η^2^ = 0.034
	Night	2.5 (2.0)	1.0 (1.8)				0.5 (0.2)	0.4 (0.4)			
**Side mirror left**	Day	2.0 (1.8)	1.3 (1.5)	F(1,43) = 3.4 p = 0.071 η^2^ = 0.074	F(1,43) = 10.3 p = 0.003 η^2^ = 0.193	F(1,43) = 0.1 p = 0.721 η^2^ = 0.003	0.5 (0.3)	0.5 (0.5)	F(1,43) = 0.6 p = 0.460 η^2^ = 0.013	F(1,43) = 0.8 p = 0.371 η^2^ = 0.019	F(1,43) = 5.1 p = 0.029 η^2^ = 0.106
	Night	1.3 (1.2)	0.7 (1.0)				0.4 (0.3)	0.3 (0.4)			
**Side mirror right**	Day	0.3 (0.6)	0.3 (0.6)	F(1,43) = 1.7 p = 0.201 η^2^ = 0.038	F(1,43) = 0.7 p = 0.403 η^2^ = 0.016	F(1,43) = 2.6 p = 0.114 η^2^ = 0.057	0.2 (0.3)	0.2 (0.4)	F(1,43) = 1.1 p = 0.304 η^2^ = 0.025	F(1,43) = 1.7 p = 0.206 η^2^ = 0.037	F(1,43) = 2.4 p = 0.145 η^2^ = 0.049
	Night	0.4 (0.7)	0.0 (0.1)				0.2 (0.3)	0.0 (0.1)			

DWELL = cumulated fixation duration/total driving task duration; GAZE DURATION = average fixation time; η^2^ refers to partial η^2^.

#### Overtaking an incoming car and car ahead

Age and light had a significant effect on the visual exploration of ROI car ahead. In addition, light had a significant effect on the ROI DWELL of environment, and side view mirror, while age affected on ROI incoming car. The interaction of light x age was associated with significant differences in side view mirror usage, duration of rear-view mirror usage, ROI DWELL incoming car and ROI DURATION car ahead (see Table 4). During overtaking older drivers focused more and longer on the ROI car ahead. This visual exploration behavior increased for both age groups at night time. Older drivers focused longer on incoming car, while younger drivers focused more on incoming car during day time and older drivers focused more on incoming car at night. Both older and younger drivers focused less on environment at night, while older drivers focused shorter on rear and side mirrors at night.

**Table 4:** **Comparison of DWELL and GAZE DURATION in addition to effects of age**, **light and interaction of age x light during the overtaking task**

		**DWELL** **[%]** **(SD)**					**DURATION** **[s]** **(SD)**				
		**Young**	**Old**	**Age**	**Light**	**Age x Light**	**Young**	**Old**	**Age**	**Light**	**Age x Light**
**Street**	Day	32.7 (7.1)	33.9 (7.1)	F(1,43) = 0.6 p = 0.447 η^2^ = 0.000	F(1,43) = 2.9 p = 0.098 η^2^ = 0.062	F(1,43) = 4.3 p = 0.044 η^2^ = 0.091	1.0 (0.3)	1.1 (0.3)	F(1,43) = 0.9 p = 0.360 η^2^ = 0.019	F(1,43) = 3.3 p = 0.077 η^2^ = 0.071	F(1,43) = 2.0 p = 0.169 η^2^ = 0.044
	Night	33.3 (10.3)	28.6 (10.8)				1.2 (0.3)	1.2 (0.4)			
**Road signs**	Day	0.4 (0.7)	0.2 (0.4)	F(1,43) = 0.2 p = 0.632 η^2^ = 0.005	F(1,43) = 0.0 p = 0.908 η^2^ = 0.000	F(1,43) = 4.0 p = 0.052 η^2^ = 0.085	0.3 (0.4)	0.2 (0.4)	F(1,40) = 0.0 p = 0.938 η^2^ = 0.000	F(1,40) = 1.0 p = 0.321 η^2^ = 0.025	F(1,40) = 2.1 p = 0.160 η^2^ = 0.049
	Night	0.2 (0.5)	0.4 (0.8)				0.2 (0.5)	0.4 (0.7)			
**Car ahead**	Day	20.1 (5.7)	23.2 (5.0)	F(1,43) = 7.1 p = 0.011 η^2^ = 0.000	F(1,43) = 60.3 p < 0.001 η^2^ = 0.584	F(1,43) = 1.0 p = 0.332 η^2^ = 0.022	0.9 (0.3)	1.1 (0.2)	F(1,43) = 8.8 p = 0.005 η^2^ = 0.170	F(1,43) = 45.7 p < 0.001 η^2^ = 0.515	F(1,43) = 4.4 p = 0.042 η^2^ = 0.093
	Night	28.8 (6.2)	34.5 (10.3)				1.3 (0.4)	1.7 (0.7)			
**Incoming car**	Day	17.0 (4.6)	16.3 (5.6)	F(1,43) = 2.5 p = 0.124 η^2^ = 0.054	F(1,43) = 0.2 p = 0.651 η^2^ = 0.005	F(1,43) = 4.9 p = 0.032 η^2^ = 0.102	2.0 (1.0)	2.6 (1.3)	F(1,42) = 4.2 p = 0.048 η^2^ = 0.190	F(1,42) = 0.2 p = 0.670 η^2^ = 0.004	F(1,42) = 0.0 p = 0.951 η^2^ = 0.000
	Night	14.7 (5.1)	19.7 (9.2)				2.1 (0.8)	2.7 (1.5)			
**Environment**	Day	3.2 (2.2)	2.2 (2.2)	F(1,43) = 2.1 p = 0.153 η^2^ = 0.047	F(1,43) = 9.1 p = 0.004 η^2^ = 0.174	F(1,43) = 0.8 p = 0.371 η^2^ = 0.019	0.7 (0.3)	0.5 (0.3)	F(1,41) = 0.9 p = 0.337 η^2^ = 0.022	F(1,41) = 0.6 p = 0.459 η^2^ = 0.013	F(1,41) = 1.7 p = 0.195 η^2^ = 0.041
	Night	1.7 (2.0)	1.4 (1.2)				0.5 (0.4)	0.5 (0.3)			
**Rear** **-view mirror**	Day	6.3 (3.6)	5.4 (3.2)	F(1,43) = 3.6 p = 0.061 η^2^ = 0.079	F(1,43) = 130.1 p < 0.001 η^2^ = 0.752	F(1,43) = 3.7 p = 0.061 η^2^ = 0.079	0.6 (0.3)	0.7 (0.2)	F(1,43) = 0.0 p = 0.972 η^2^ = 0.000	F(1,43) = 4.1 p = 0.050 η^2^ = 0.086	F(1,43) = 4.9 p = 0.033 η^2^ = 0.101
	Night	4.6 (3.2)	2.3 (2.0)				0.6 (0.2)	0.6 (0.3)			
**Side mirror left**	Day	2.5 (1.7)	2.6 (1.6)	F(1,43) = 0.8 p = 0.371 η^2^ = 0.019	F(1,43) = 9.9 p = 0.003 η^2^ = 0.187	F(1,43) = 5.3 p = 0.026 η^2^ = 0.003	0.7 (0.3)	0.7 (0.3)	F(1,43) = 0.8 p = 0.372 η^2^ = 0.019	F(1,43) = 3.8 p = 0.057 η^2^ = 0.082	F(1,43) = 0.0 p = 0.973 η^2^ = 0.067
	Night	2.4 (1.5)	1.5 (1.4)				0.6 (0.3)	0.6 (0.3)			
**Side mirror right**	Day	0.5 (1.1)	0.5 (0.8)	F(1,43) = 0.4 p = 0.536 η^2^ = 0.006	F(1,43) = 1.9 p = 0.171 η^2^ = 0.043	F(1,43) = 0.8 p = 0.376 η^2^ = 0.018	0.2 (0.4)	0.3 (0.4)	F(1,43) = 0.0 p = 0.987 η^2^ = 0.000	F(1,43) = 1.35 p = 0.251 η^2^ = 0.030	F(1,43) = 0.4 p = 0.530 η^2^ = 0.009
	Night	0.4 (0.8)	0.2 (0.5)				0.2 (0.3)	0.2 (0.4)			

DWELL = cumulated fixation duration/total driving task duration; GAZE DURATION = average fixation time; η^2^ refers to partial η^2^.

#### Avoiding collision with a parked car

Light had a significant effect on the visual exploration of ROI street, road signs and parked car, while age had an effect on exploration of ROI parked car and side view mirror. An interaction of light x age was found for gaze duration on ROI parked car (see Table 5). Generally, older drivers focused more on the parked car, while younger drivers focused longer on parked car during daytime and older drivers focused more on parked car at night. The visual exploration for road signs increased while the focus on the street was shorter during night time. The usage of side view (right) mirror was almost none for the older drivers during this task.

**Table 5:** **Comparison of DWELL and DURATION in addition to effects of age**, **light and interaction of age x light while avoiding collision with a parked car**

		**DWELL** **[%]** **(SD)**					**DURATION** **[s]** **(SD)**				
		**Young**	**Old**	**Age**	**Light**	**Age x light**	**Young**	**Old**	**Age**	**Light**	**Age x light**
**Street**	Day	26.3 (11.9)	30.5 (15.6)	F(1,43) = 0.0 p = 0.901 η^2^ = 0.000	F(1,43) = 3.1 p = 0.085 η^2^ = 0.067	F(1,43) = 2.4 p = 0.125 η^2^ = 0.054	1.0 (0.8)	1.3 (0.7)	F(1,39) = 0.2 p = 0.638 η^2^ = 0.006	F(1,39) = 5.3 p = 0.027 η^2^ = 0.119	F(1,39) = 3.7 p = 0.063 η^2^ = 0.086
	Night	25.8 (13.1)	22.0 (12.6)				1.0 (0.6)	0.8 (0.4)			
**Road signs**	Day	0.0 (0.0)	0.0 (0.0)	F(1,43) = 0.6 p = 0.427 η^2^ = 0.015	F(1,43) = 38.4 p < 0.001 η^2^ = 0.472	F(1,43) = 0.6 p = 0.427 η^2^ = 0.015	0.0 (0.0)	0.0 (0.0)	F(1,37) = 1.3 p = 0.268 η^2^ = 0.033	F(1,37) = 36.2 p < 0.001 η^2^ = 0.494	F(1,37) = 1.3 p = 0.268 η^2^ = 0.033
	Night	6.7 (6.3)	5.1 (6.5)				0.6 (0.6)	0.4 (0.5)			
**Parked car**	Day	50.4 (11.3)	51.6 (17.7)	F(1,43) = 4.6 p = 0.039 η^2^ = 0.096	F(1,43) = 0.3 p = 0.573 η^2^ = 0.007	F(1,43) = 6.6 p = 0.014 η^2^ = 0.132	1.6 (0.7)	1.6 (0.6)	F(1,42) = 6.4 p = 0.014 η^2^ = 0.136	F(1,42) = 12.9 p = 0.001 η^2^ = 0.234	F(1,42) = 9.32 p = 0.004 η^2^ = 0.182
	Night	42.5 (12.1)	56.6 (17.1)				1.6 (0.4)	2.6 (1.4)			
**Environment**	Day	1.5 (2.5)	0.9 (3.0)	F(1,43) = 0.3 p = 0.590 η^2^ = 0.007	F(1,43) = 2.7 p = 0.108 η^2^ = 0.059	F(1,43) = 0.5 p = 0.472 η^2^ = 0.012	0.2 (0.2)	0.1 (0.2)	F(1,43) = 0.8 p = 0.380 η^2^ = 0.018	F(1,43) = 1.1 p = 0.312 η^2^ = 0.024	F(1,43) = 1.1 p = 0.312 η^2^ = 0.024
	Night	0.5 (1.6)	0.5 (1.8)				0.1 (0.2)	0.1 (0.2)			
**Rear** **-view mirror**	Day	4.2 (4.5)	2.3 (3.8)	F(1,43) = 3.4 p = 0.071 η^2^ = 0.074	F(1,43) = 0.6 p = 0.437 η^2^ = 0.014	F(1,43) = 0.1 p = 0.825 η^2^ = 0.001	0.3 (0.3)	0.2 (0.4)	F(1,43) = 1.7 p = 0.199 η^2^ = 0.042	F(1,43) = 1.1 p = 0.299 η^2^ = 0.028	F(1,43) = 0.3 p = 0.616 η^2^ = 0.007
	Night	3.5 (4.3)	1.9 (3.3)				0.4 (0.5)	0.3 (0.4)			
**Side mirror left**	Day	3.9 (3.9)	3.0 (4.3)	F(1,43) = 1.7 p = 0.201 η^2^ = 0.038	F(1,43) = 2.1 p = 0.157 η^2^ = 0.046	F(1,43) = 0.1 p = 0.706 η^2^ = 0.003	0.4 (0.4)	0.3 (0.4)	F(1,42) = 2.1 p = 0.159 η^2^ = 0.047	F(1,42) = 0.3 p = 0.569 η^2^ = 0.008	F(1,42) = 3.0 p = 0.091 η^2^ = 0.067
	Night	3.2 (2.0)	1.9 (3.4)				0.5 (0.4)	0.3 (0.4)			
**Side mirror right**	Day	0.7 (1.9)	0.0 (0.0)	F(1,43) = 5.7 p = 0.022 η^2^ = 0.116	F(1,43) = 0.0 p = 0.889 η^2^ = 0.000	F(1,43) = 0.3 p = 0.573 η^2^ = 0.007	0.1 (0.3)	0.0 (0.0)	F(1,43) = 5.8 p = 0.021 η^2^ = 0.118	F(1,43) = 0.2 p = 0.642 η^2^ = 0.005	F(1,43) = 0.0 p = 0.939 η^2^ = 0.000
	Night	0.6 (1.2)	0.1 (0.5)				0.1 (0.3)	0.0 (0.1)			

DWELL = cumulated fixation duration/total driving task duration; GAZE DURATION = average fixation time; η^2^ refers to partial η^2^.

### Driving performance

Age had a significant main effect on driving speed, driving speed variance, lane-keeping, and lane-keeping variance while light conditions had a significant main effect on lane-keeping (see Table 6). No significant effect of interaction light x age was found for the driving speed and lane-keeping performances.

**Table 6:** **Group differences and effect of age**, **light and interaction of light x age on driving performance** (**measured on a straight stretch**)

		**Young** **(N** ** = 28)**	**Old** **(N** ** = 25)**	**Significance**		
				**Age**	**Light**	**Light x Age**
**Driving speed [km/h] (SD)**	Day	82.0 (2.4)	78.5 (3.1)	F(1,51) = 18.8 p < 0.001 η^2^ = 0.269	F(1,51) = 2.1 p = 0.162 η^2^ = 0.038	F(1,51) = 1.2 p = 0.278 η^2^ = 0.023
	Night	82.1 (2.4)	79.5 (3.8)			
**Driving speed variance [km/h] (SD)**	Day	2.4 (2.5)	12.7 (12.5)	F(1,51) = 30.4 p < 0.001 η^2^ = 0.373	F(1,51) = 2.8 p = 0.099 η^2^ = 0.052	F(1,51) = 1.2 p = 0.279 η^2^ = 0.023
	Night	4.0 (5.4)	20.0 (26.4)			
**Lane-keeping [degree] (SD)**	Day	0.4 (0.2)	0.7 (0.3)	F(1,51) = 19.2 p < 0.001 η^2^ = 0.274	F(1,51) = 10.4 p = 0.002 η^2^ = 0.169	F(1,51) = 1.5 p = 0.229 η^2^ = 0.028
	Night	0.5 (0.3)	0.9 (0.4)			
**Lane-keeping variance [degree] (SD)**	Day	0.2 (0.1)	0.6 (0.7)	F(1,51) = 13.4 p = 0.001 η^2^ = 0.209	F(1,51) = 0.8 p = 0.384 η^2^ = 0.015	F(1,51) = 0.1 p = 0.705 η^2^ = 0.003
	Night	0.2 (0.2)	0.7 (1.1)			

η^2^ refers to partial η^2^.

Although there was a significant difference in driving speed between the two groups, the speed of both age groups was close to the speed limit. Speed variability was smaller in the younger age group in both light conditions compared to the older age group. The speed variability for older drivers further increased during night driving. In addition to the higher variability of driving speed, older drivers showed a lower precision in lane-keeping compared to the younger drivers. The precision in lane-keeping degraded for both age groups in the night conditions.

## Discussion

The primary aim of this study was to analyze the age-dependent effect on visual exploration during simulated day- and night driving. We showed that older drivers had significant longer mean fixation durations and focused longer on the task relevant ROI for specific tasks such as street for straight driving, car ahead and incoming car during overtaking and parked car while avoiding collision. Younger drivers focused more on the rear-view mirror during the straight and narrow lane driving task. Night driving had a significant effect on mean fixation durations and on visual exploration behavior for relevant ROI for narrow lane and overtaking. The focus on rear-view and side mirror was reduced at night for all tasks, while a significant focus reduction on environment was observed for the overtaking task. Older drivers had more heterogeneous driving with more speed variability and lower lane-keeping precision compared to younger drivers. Night driving worsened older drivers’ speed variance and lane-keeping precision, but had no effect on driving speed and lane-keeping in the younger age group.

Only a few studies have assigned visual fixations to pre-defined ROIs during driving, with the exception of specific tasks such as curve driving or intersections [15,16,18,19]. On straight rural roads during the day, drivers spent 65.9% of the time focusing on the road, 8.0% on the scenery, and 3.7% on the rearview mirror [40]. In our straight driving task, subjects focused to a similar extent on the street (young: 48.2%, old: 55.5%), less on the environment (young: 4.2%, old: 1.5%), and similar on rear-view mirror (young: 6.0%, old: 3.3%) during daylight driving. The different values for the environment can be explained by the different driving routes (rural roads vs. motorway). With respect to age effects in the straight driving task, older drivers focused more on central parts of the driving scene (street) compared with younger drivers. This is in accordance with a study conducted by Maltz et al. [24], which showed that older subjects focus on a smaller subset of areas compared to younger subjects whose exploration is more evenly distributed. However, Underwood et al. [41] found no age-related decline in the search of the scene when detecting hazards. The different results between the studies could be explained by different study designs (photographs of actual traffic scenes vs. film clips with driving situations vs. driving simulator task), different definitions of age groups, and small sample sizes. Existing literature reports that drivers spend more time looking straight ahead neglecting peripheral regions and mirrors when workload increases [42,43]. By applying this to our results, we suggest that the higher attention to the street ahead neglecting peripheral regions and mirrors by older subjects is due to the higher workload caused for older drivers compared to younger drivers. The higher workload to avoid collision explains why older subjects in our study ignored the side view right mirror. Checking the rearview mirror has been used as a measure for attention paid to other traffic [44,45]. Our study revealed that younger drivers check the mirrors more frequently, which is in line with other literatures [16,18,24]. On the one hand, crashes occurring while changing lanes are more common among older drivers [46], which might be explained by the reduced frequency of checking mirrors of older drivers [47]. On the other hand, active training with driving-specific feedback increases older drivers’ frequency of visual inspections of mirrors and blind spot prior to lane changes [48].

During night driving, our results showed a significantly higher ROI DWELL on the task relevant ROIs for both age groups and a lower ROI DWELL on environment in younger drivers, which is consistent with existing literature [8,49,50]. Night driving is more demanding, which increases workload and thus results in a reduced horizontal spread. Our results further showed reduced mirror checking during night driving, which can be explained by the increased workload [43]. Another explanation could be the low saliency in the mirrors which could have resulted in giving the drivers no reason to check the mirrors. More traffic with more car headlight would have resulted in a higher saliency in the rearview and might have caught the driver’s attention to the mirrors more frequently.

The mean fixation durations found in this study are perfectly in line with the range of 200 ms to 350 ms for mean fixation durations reported by Green [51]. Our results of longer mean fixation durations for night driving and for older drivers are in line with results reported in literature [20,23,49,50,52], but in contrast to others who reported no age-differences [24] or longer durations for younger drivers [40]. These differences in results can be explained by different study designs (e.g., rural vs. urban roads) as the least visually complex rural roads attract the longest mean fixation durations while visually complex urban roads lead to shorter mean fixation durations [53]. During night driving, less visual information is available, which may lead to the longer fixation durations we obtained during night driving. Regarding the age-dependent differences, longer mean fixation durations indicate that older drivers require longer time to extract the meaning of elements in the environment [52] and support earlier findings that older drivers typically look at an object longer and more frequently to extract the same information from it as younger drivers [54].

Our findings that older drivers showed slower speed and more heterogeneous driving speed with worse lane-keeping behavior is consistent with literature [27,28,55]. The heterogeneous driving behavior seems to be linked to car crashes [55]. A significant, but small age-related difference in driving speed was found between the two age groups. However, the speed of both age groups was around the speed limit with younger subjects’ speed slightly higher than the speed limit. In low luminance, previous research has revealed that older drivers exhibit a progressive degradation of steering accuracy, not found with younger drivers [29]. This finding is perfectly in line with our results. However, younger drivers’ speed and lane-keeping behavior seem to be rather unaffected by light conditions.

Driving simulators can assist in understanding the problems of night driving. The levels of illumination derived from headlamps, oncoming vehicles as well as those from ambient lighting sources have an impact on the visual performance of the driver [56]. The luminance values mentioned here are measured above the carriageway from a point halfway across the drivers section. However, this study has a few limitations. First, people might react differently in driving simulators since there is no risk of collision or physical harm [57]. Wearing a head mounted eye tracker system may restrict eye movements and head movements which may be confused with imposed restrictions by older subjects. Reflections from the infrared beam from eyeglass lenses and frames may interfere with obtaining a reliable corneal reflection; loss of eye movement data can result. Gaze tracking measures only central vision and not peripheral vision while peripheral vision also contributes during the driving task (e.g. for object detection). However, using a head-mounted gaze tracker to measure visual exploration behavior during driving is commonly used in research. Many older drivers avoid driving at night and this makes them less secure in night driving tests. However, in our study, only one older subject did not drive at night during the last three months. The mean weekly mileage of the older drivers was almost half that of the young participants which could indicate a driver experience effect in our observations. On the other side, older drivers have a longer history and experience in driving than their younger counterparts.

## Conclusions

In conclusion, our findings show effects of age and light conditions on visual exploration behavior. Older drivers have a narrowed visual exploration behavior during simulated driving on a motorway, especially during night driving. When applying the workload hypothesis, we conclude that older drivers are more challenged than younger drivers by simulated driving, especially during night driving. This can also be supported by the more heterogeneous driving speed and lane-keeping behavior found in older drivers.
